# Hypothalamic-Pituitary-Adrenal Axis Activity and Metabolic Disorders in Kidney Transplant Recipients on Long-Term Glucocorticoid Therapy

**DOI:** 10.3390/jcm13226712

**Published:** 2024-11-08

**Authors:** Stathis Tsiakas, Anna Angelousi, Vassiliki Benetou, Philippos Orfanos, Efstathios Xagas, John Boletis, Smaragdi Marinaki

**Affiliations:** 1 Department of Nephrology and Renal Transplantation, Medical School, National and Kapodistrian University of Athens, Laiko Hospital, 11527 Athens, Greece; stsiakas@med.uoa.gr (S.T.); exagas@med.uoa.gr (E.X.); inboletis@med.uoa.gr (J.B.); smarinak@med.uoa.gr (S.M.); 2 Unit of Endocrinology, First Department of Internal Medicine, Laikon Hospital, Center of Excellence of Endocrine Tumours, National and Kapodistrian University of Athens, 11527 Athens, Greece; 3 Department of Hygiene, Epidemiology and Medical Statistics, Medical School, National and Kapodistrian University of Athens, 11527 Athens, Greece; vbenetou@med.uoa.gr (V.B.); phorfanos@med.uoa.gr (P.O.)

**Keywords:** HPA axis suppression, kidney transplantation, morning cortisol, ACTH, metabolic disorders

## Abstract

**Background/Objectives:** Glucocorticoids are commonly used for maintenance immunosuppressive therapy in kidney transplant recipients (KTRs). We aimed to investigate the prevalence of hypothalamic-pituitary-adrenal (HPA) axis suppression and its association with metabolic disorders in stable KTRs on low-dose glucocorticoids. **Methods**: This cross-sectional study included adult KTRs on low-dose glucocorticoids. HPA axis suppression was defined as baseline morning cortisol < 5 μg/dL. Adrenocorticotropic hormone (ACTH), dehydroepiandrosterone-sulphate (DHEAS) and 24 h urinary free cortisol (UFC) levels were also assessed. Examined metabolic disorders included hypertension, dyslipidemia, central obesity and post-transplant diabetes mellitus (PTDM). **Results**: Eighty adult KTRs with a median 57 months (IQR 24–102) since transplantation were included in the study. The mean prednisolone dose was 5.0 ± 1.3 mg/day. Baseline cortisol < 5.0 μg/dL was observed in 27.5% of the KTRs. Participants with baseline cortisol < 5.0 μg/dL were older (55.1 vs. 47.4 years, *p* = 0.023) and had had a transplant for a longer time (101.4 vs. 67.0 months, *p* = 0.043), compared with the rest of the cohort. Baseline cortisol correlated positively with ACTH (rho = 0.544, *p* < 0.001), DHEAS (rho:0.459, *p* < 0.001) and UFC (rho: 0.377, *p* = 0.002). The area under the receiver-operating characteristic curve for ACTH as a predictor of baseline cortisol > 5.0 μg/dL was 0.79 [95% confidence interval (CI): 0.68–0.89]. After adjustment for covariates, HPA axis suppression was not associated with the examined metabolic disorders. **Conclusions**: Our study showed that stable KTRs on chronic low-dose glucocorticoids exhibited an increased prevalence of HPA axis suppression. ACTH may serve as a surrogate biomarker for HPA axis activity in this population. Further research could evaluate the association of glucocorticoid-induced HPA axis inhibition with metabolic disorders.

## 1. Introduction

Glucocorticoids remain a part of maintenance immunosuppressive therapy in most kidney transplant recipients (KTRs) [1]. While they are effective in preventing renal graft rejection, long-term glucocorticoids are generally related to a series of adverse metabolic events, potentially increasing cardiovascular risk and mortality [2,3,4,5]. Advances in immunosuppression may allow avoidance or withdrawal of steroids in many KTRs; however, a significantly increased risk of acute rejection was observed with these regimens compared to maintenance glucocorticoid therapy [5]. Efforts to minimize the effects of chronic steroid exposure have led to the implementation of low-dose glucocorticoid medications (e.g., prednisolone 2.5–7.5 mg/day) for long-term maintenance immunosuppression in kidney transplantation [6,7,8].

Glucocorticoids exert negative feedback on hypothalamic-pituitary-adrenal (HPA) axis activity, inhibiting the release of corticotropin-releasing hormone by the hypothalamus and adrenocorticotropic hormone (ACTH) by the anterior pituitary gland. Suppressed ACTH levels lead to atrophy of the adrenal cortex and reduced cortisol production [9]. The risk of developing adrenal insufficiency (AI) after steroid discontinuation varies, depending on the glucocorticoid potency and dosage, duration of treatment and individual susceptibility [10,11,12]. Any route of administration and any dose surpassing the physiologic daily dose equivalent (4–6 mg prednisolone) to average endogenous cortisol production could result in HPA axis suppression [10]. Nevertheless, the likelihood of AI is difficult to predict even in physiological dose equivalents due to considerable inter-individual variability in glucocorticoid pharmacodynamics and pharmacokinetics [13,14,15].

HPA axis suppression is common in kidney transplantation. A systematic review and meta-analysis showed a pooled absolute risk of 56.2% for AI in glucocorticoid-treated KTRs [10]. Included studies, though, displayed high heterogeneity in the used serum cortisol assays and HPA axis evaluation tests, as well as in the mean prednisolone dose intake. Clinical studies evaluating HPA axis activity from previous decades involved KTRs receiving mostly supraphysiologic glucocorticoid doses, up to 20 mg of daily prednisolone, which are not currently used as maintenance immunosuppression [16,17,18,19,20,21,22]. Recent cross-sectional studies, including one in a pediatric population, reported a prevalence of AI between 31.3% and 72.0% in KTRs on low-dose maintenance prednisolone [23,24,25,26]. Adrenal function was primarily assessed using a short Synacthen test (SST) conducted at least 24 h after the last glucocorticoid dose. Glucocorticoid-induced HPA axis suppression was linked to a higher daily prednisolone dose and a greater cumulative steroid exposure in two of these studies [24,26].

Prolonged glucocorticoid treatment, even in relatively low doses, may increase the incidence of adverse metabolic events. Most data come from large cohorts of rheumatoid arthritis patients, in which chronic glucocorticoid use was associated with higher rates of serious metabolic outcomes and all-cause mortality [27,28]. Steroid avoidance or withdrawal protocols in kidney transplantation have led to significant reductions in the risk of hypertension, new-onset diabetes mellitus and dyslipidemia although these benefits were accompanied by an increased risk of acute rejection episodes [5]. Secondary HPA axis suppression is frequently observed in long-term steroid-treated patients and may be associated with an increased incidence of cardiovascular risk factors and mortality. A dose-dependent risk of adrenal dysfunction and mortality was shown in a large cohort of patients with chronic inflammatory diseases receiving oral glucocorticoids [29]. Furthermore, a Dutch cross-sectional study evaluating 24 h urinary free cortisol (UFC) excretion in a large cohort of stable KTRs found a significant relationship between suppressed UFC levels and the prevalence of metabolic syndrome factors [30].

The primary objective of this study was to assess the frequency of HPA axis suppression in KTRs on low-dose glucocorticoid therapy. A secondary analysis aimed to investigate whether an association exists between HPA axis suppression and significant metabolic disorders, including hypertension, dyslipidemia, central obesity and post-transplant diabetes mellitus (PTDM).

## 2. Materials and Methods

### 2.1. Study Participants

Participants of the study were recruited from the outpatient clinic of the Department of Nephrology and Renal Transplantation at “Laiko” General Hospital of Athens, Greece. Eligible patients were adult KTRs, with at least 1 year of post-transplant follow-up, an estimated glomerular filtration rate (eGFR) ≥ 60 mL/min/1.73 m^2^ by the Chronic Kidney Disease Epidemiology Collaboration (CKD-EPI) equation [31] and a stable immunosuppressive regimen including low-dose corticosteroids. Exclusion criteria consisted of immunosuppressive treatment modification in the last 3 months, an acute rejection episode in the last year and a history of AI or hypothalamic-pituitary pathology. We excluded participants with moderate or severe renal impairment (eGFR < 60 mL/min/1.73 m^2^) due to potential changes of free prednisolone clearance [32].

The protocol of the study was approved by the Ethics Review Board of “Laiko” General Hospital of Athens, Greece (No 5651/3 April 2020). All participants provided signed informed consent before their enrollment in the study. The study was conducted in accordance with the principles of the Declaration of Helsinki.

### 2.2. Data Collection and Study Procedures

In this cross-sectional study, all participants were evaluated during a single, scheduled morning visit at the transplant outpatient clinic between March 2021 and February 2022. Their medical record was reviewed prior to the visit in order to assess the eligibility criteria for the study. Demographic and anthropometric characteristics, comorbidities and current medication, as well as biochemical parameters, were collected for each participant and recorded in a purpose-built electronic datasheet.

Participants were asked to withhold their morning oral glucocorticoid medication and provide us with a 24 h urine sample. Careful instructions for the 24 h urine collection were given prior to their visit to ensure a credible specimen. Fasting laboratory workup, including serum lipid profile [low-density lipoprotein-cholesterol (LDL-C), high-density lipoprotein-cholesterol (HDL-C), triglycerides] and glucose and hemoglobin A1c (HbA1c) levels, was performed as per routine clinical practice, and blood samples for the evaluation of HPA axis function were collected for the purpose of the study. Examined hormonal biomarkers included morning (8:00 am) serum cortisol, serum dehydroepiandrosterone-sulphate (DHEAS), plasma ACTH and 24 h UFC levels using in-hospital assays. Cortisol and UFC levels were measured with the electro chemiluminescent bridging immunoassay (ECLIA) [Cobas 8000 e801, Roche Diagnostics, Mannheim, Germany; Hitachi, Tokyo, Japan; intra-assay coefficients of variation (CV) < 3.9% and inter-assay CV < 3.8%], ACTH with the chemiluminescent assay (Liaison, DiaSorin, Saluggia, Italy; intra-assay CV 4.3–7.5% and inter-assay CV 10–14.5%) and DHEAS with the chemiluminescent competitive binding immunoenzymatic assay (Beckman Coulter, Brea, CA, USA; intra-assay CV 1.6–8.3% and inter-assay CV 3.7–11.3%).

The HPA axis activity evaluation was based on measurements of baseline morning serum cortisol levels, along with concurrent assessments of ACTH, DHEAS and UFC levels 24 h after the last dose of prednisolone. HPA axis suppression was defined as a morning (8:00 a.m.) serum cortisol < 5 μg/dL, as suggested by the European Society of Endocrinology and Endocrine Society Joint Clinical Guideline [15]. Blood pressure was measured by a healthcare worker in the office, and the American College of Cardiology/American Heart Association guidelines were applied for the diagnosis of hypertension or the use of any antihypertensive medication [33]. Dyslipidemia was identified according to the International Diabetes Federation criteria [34] and central obesity according to World Health Organization expert consultation report [35]. PTDM was defined as the detection of diabetes after transplantation, irrespective of the timing of diagnosis or whether it was present but unrecognized prior to transplantation [36]. The American Diabetes Association criteria or the initiation of anti-diabetic treatment after transplantation was used for the diagnosis of PTDM [37].

### 2.3. Statistical Analysis

Data were expressed as means and standard deviations or medians and interquartile ranges (IQR), depending on the data distribution. Categorical variables were presented as absolute values and percentages. Continuous variables were compared using the two independent samples *t*-test or Mann–Whitney U test, depending on the distribution normality. Chi-squared tests were applied to examine the association between categorical variables. Spearman’s correlation co-efficient was used to assess the strength and direction of the relationship between two continuous variables. A receiver operating characteristics (ROC) analysis was performed to assess the ACTH level as a potential predictor of HPA axis suppression, with an area under the curve (AUC) of 1.0 representing perfect discrimination. An ACTH cut-off value was introduced using the Youden index, and its sensitivity and specificity were calculated. Simple and multiple logistic regression analyses, adjusting for age, sex, body mass index (BMI), eGFR, tacrolimus intake and prednisolone dose were used to evaluate the association between HPA axis suppression and metabolic parameters. *p*-values ≤ 0.05 were considered statistically significant. Statistical analysis was performed using IBM SPSS Statistics Version 23 (SPSS Inc., Chicago, IL, USA).

## 3. Results

### 3.1. Demographic and Clinical Characteristics of the Participants

A total of 80 adult KTRs with a mean age of 49.6 (±14.8) years were included in the study. Most of them were male, 68.8% (*n* = 55), and had undergone a living donor kidney transplant, 51.3% (*n* = 41). Participants’ primary kidney disease was mostly unknown, 32.5% (*n* = 26), followed by glomerulonephritis, 25.0% (*n* = 20), and adult polycystic kidney disease, 15.0% (*n* = 12), in order of prevalence. The mean dialysis vintage was 4.9 ± 3.8 years. The mean serum creatinine level of the KTRs was 1.14 ± 0.20 mg/dL, corresponding to a mean eGFR of 71.1 mL/min/1.73 m^2^. Immunosuppressive treatment was primarily tacrolimus-based [85.0%, (*n* = 68)]. Cyclosporine and everolimus were used by 5.0% (*n* = 4) and 15.0% (*n* = 12) of the participants, respectively. All KTRs were on a stable glucocorticoid regimen with a mean prednisolone dose of 5.0 ± 1.3 mg/dL. The median time from transplantation was 57 (IQR 24–102) months.

### 3.2. Morning Serum Cortisol and Its Correlation with Other Adrenal Biomarkers

The mean morning cortisol level was 9.0 ± 5.0 μg/dL. A total of 22 KTRs (27.5%) had a morning cortisol level < 5.0 μg/dL, 31 KTRs (38.8%) had a morning cortisol level between 5 and 10 μg/dL, and 27 KTRs (33.7%) had a level above 10 μg/dL. Participants with suppressed morning cortisol levels (<5.0 μg/dL) were older (55.1 vs. 47.4 years, *p* = 0.023) than the rest of the cohort and had the kidney transplant for a longer period (101.4 vs. 67.0 months, *p* = 0.043). No significant difference between the two groups (participants with suppressed morning cortisol levels < 5.0 μg/dL vs. all others) was found with regard to sex, BMI, or dialysis vintage. The demographic and clinical characteristics among all participants and by baseline cortisol levels are depicted in detail in Table 1.

Participants with decreased morning cortisol levels (<5.0 μg/dL) also had lower levels of ACTH (10.7 vs. 20.5, *p* < 0.001) pg/mL, DHEAS (27.2 vs. 80.7, *p* = 0.001) μg/mL, and UFC (6.5 vs. 15.5, *p* = 0.002) μg/24 h. The adrenal biomarkers levels of all participants and by baseline cortisol levels are shown in Table 2. Spearman’s correlation test displayed a stronger positive relation of morning cortisol levels with ACTH (rho: 0.544, *p* < 0.001), followed by DHEAS (rho: 0.459, *p* < 0.001) and UFC (rho: 0.377, *p* = 0.002).

**Figure 1:**
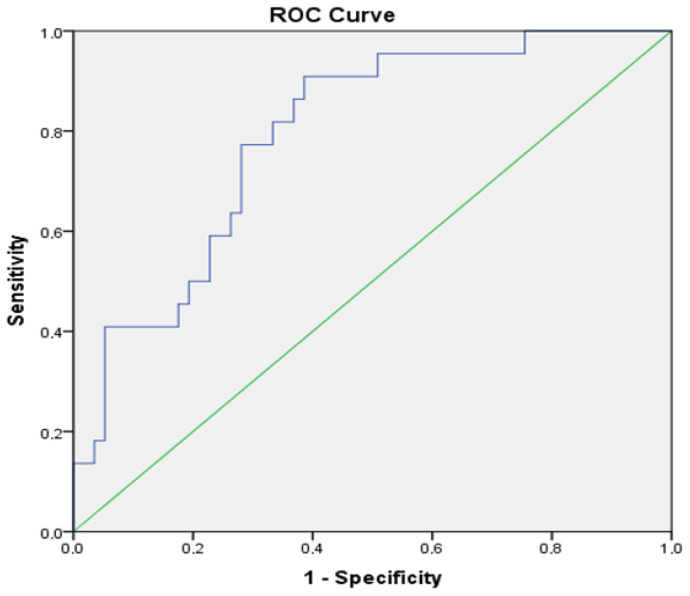
ROC curve demonstrating the performance of ACTH as a predicting tool for a morning cortisol level ≥ 5 μg/dL (AUC: 0.79, 95% CI: 0.68–0.89).

ROC analysis showed that ACTH [AUC: 0.79, 95% confidence interval (CI): 0.68–0.89] could be used to predict HPA axis suppression in steroid-treated KTRs (Figure 1). An ACTH value of 12.8 pg/mL predicted a baseline cortisol level > 5.0 μg/dL with a sensitivity of 77.3% and a specificity of 71.9% in our cohort.

### 3.3. Morning Serum Cortisol and Its Association with Adverse Metabolic Factors

Hypertension, dyslipidemia, central obesity and PTDM were observed in 72.5% (*n* = 58), 62.5% (*n* = 50), 45.0% (*n* = 36) and 13.8% (*n* = 11) of the included KTRs, respectively. Hypertension was more prevalent among participants with suppressed morning cortisol levels (<5.0 μg/dL), compared with the rest of the cohort (95.1% vs. 64.9%, *p* = 0.006). No difference was found in serum triglycerides, HDL-C and LDL-C levels between the two groups. Central obesity was more frequent among KTRs with suppressed morning cortisol (*p* = 0.039). A slight increase in fasting glucose (107.0 vs. 101.2 mg/dL, *p* = 0.470) and HbA1c levels (6.0 vs. 5.6%, *p* = 0.140) was exhibited in KTRs with potential glucocorticoid-induced HPA axis suppression. The metabolic disorders among all participants and by baseline cortisol levels are depicted in Table 3.

Simple logistic regression analysis showed that suppressed morning cortisol levels were associated with hypertension [Odds ratio (OR): 11.35, 95% CI: 1.42–90.72] and central obesity (OR: 2.86, 95% CI: 1.03–7.92). After controlling for other covariates, the associations between suppressed morning cortisol and both hypertension and central obesity were no longer significant. The participants’ association of HPA axis suppression and metabolic factors is displayed in detail in Table 4.

The fully adjusted multiple model was adjusted for age, sex, body mass index (BMI), estimated glomerular filtration rate (eGFR), tacrolimus intake and prednisolone dose.

## 4. Discussion

This cross-sectional study investigated HPA axis suppression in stable KTRs with preserved renal function who are on low-dose glucocorticoid therapy. HPA axis assessment was based on morning serum cortisol levels according to current clinical guidelines [15]. The prevalence of HPA axis suppression was 27.5% in our cohort, despite participants receiving a maintenance prednisolone dose within the physiological equivalent range (4–6 mg per day). An additional 38.8% of the participants had a baseline cortisol level between 5 and 10 μg/dL, suggesting potential HPA axis recovery, while only 33.7% had a level above 10 μg/dL, indicating preserved adrenal function. Participants with a suppressed HPA axis activity were older and had had a transplant for a longer time, possibly reflecting a higher glucocorticoid cumulative exposure. Serum morning cortisol correlated positively with ACTH, DHEAS and UFC levels in our cohort. Hypertension and central obesity were observed more frequently in KTRs with suppressed HPA axis. However, after adjusting for potential confounding factors, no significant association was found between glucocorticoid-induced HPA axis inhibition and these metabolic parameters.

The prevalence of reported glucocorticoid-induced HPA axis suppression (27.5%) in our study was slightly lower than the range observed in other similar studies (31.3% to 72.0%) with low-dose glucocorticoid-treated KTRs [23,24,25,26]. Possible explanations for this difference are the different assessment assays used for the evaluation of HPA axis activity and the heterogeneity in the definitions of adrenal suppression across studies. For instance, in a recent study by Tomkins et al., an SST was conducted to assess the adrenal function, and a peak cortisol response to SST > 430 nmol/L (15.5 μg/dL) was regarded as a normal response; failure to achieve a normal response was defined as AI [26]. Patients who failed the SST in the aforementioned study had a median baseline cortisol level of 5.1 μg/dL (IQR 3.2–7.6). Using a predefined cut-off value of 5 μg/dL for defining HPA axis suppression, as we did in our study, would result in a sufficient number of these KTRs being classified as not having HPA axis inhibition. An earlier meta-analysis in steroid-treated patients reported a particularly elevated pooled absolute risk (56.2%) for AI in KTRs, second only to patients with hematologic malignancies [10]. This meta-analysis also included studies with KTRs receiving higher average doses of prednisolone compared to those in our study, thus potentially explaining the increased prevalence of reported AI. Renal impairment may lead to reduced clearance of free prednisolone, likely contributing to the glucocorticoid-induced HPA axis suppression observed in other studies [32,38]. To our knowledge, this is the first study to include only KTRs with well-preserved renal graft function (eGFR ≥ 60 mL/min/1.73 m^2^) to minimize this effect. Lastly, interactions between prednisolone and various immunosuppressive agents or other medications, as well as inter-individual differences, should not be overlooked as potential factors for the diverse prevalence of HPA axis suppression between studies [13,39].

Baseline morning cortisol levels can be used as a first-line screening tool to predict AI in patients on a physiological daily dose equivalent, aiming to discontinue glucocorticoid therapy, according to the recent European Society of Endocrinology and Endocrine Society Joint Clinical Guideline [15]. A baseline cortisol level below 5.0 μg/dL (150 nmol/L) suggests the need to maintain glucocorticoid therapy, with a follow-up test recommended after a few months. Conversely, baseline cortisol levels above 10.0 μg/dL (300 nmol/L) indicate recovery of the intrinsic HPA axis, allowing for the safe discontinuation of glucocorticoids. According to the authors, dynamic testing for diagnosing AI could be considered only for patients with persistent cortisol levels between 5.0 μg/dL and 10.0 μg/dL. Morning serum cortisol was evaluated as a diagnostic tool after validation with a dynamic 250 μg ACTH test [40,41,42]. Sbardella et al. showed that a baseline cortisol level ≤ 4.5 μg/dL (124 nmol/L) had a 100% sensitivity rate for predicting dynamic testing failure [40]. Accordingly, in the study by Debono et al., a baseline cortisol level < 5.5 μg/dL (152 nmol/L) demonstrated a positive predictive value of 95% for diagnosing glucocorticoid-induced AI [15,42]. In kidney transplantation, a baseline morning cortisol level above 10.4 μg/dL (288 nmol/L) predicted a normal SST with 100% specificity and 70% sensitivity, limiting the need for dynamic testing in steroid-treated patients [26].

During glucocorticoid treatment, ACTH secretion remains suppressed due to CRH inhibition. Gradual tapering to physiological equivalent doses can lead to an increase in ACTH and recovery in cortisol production [43]. Baseline ACTH levels in the study by Tomkins et al. were significantly lower in KTRs with AI [26], similar to our findings. ACTH displayed a significant correlation with morning cortisol in our cohort, demonstrating a potential utility in predicting a cortisol level above 5.0 μg/dL. Additional hormonal biomarkers, including DHEAS and UFC levels, were used to assess HPA axis activity in this study sample. DHEAS is primarily excreted by the adrenal glands under the regulation of ACTH and has a long half-life, allowing for a rapid evaluation of HPA axis [44,45,46]. An earlier study found that DHEAS was a suitable marker for excluding HPA axis suppression in asthmatic children on inhaled glucocorticoids [47]. UFC measurements provide an integrated measure of cortisol secretion and can be used as a surrogate for HPA axis function [48,49]. Exogenous glucocorticoids lead to suppressed UFC excretion [50]. Due to the influence of GFR on UFC levels [49], we included only KTRs with an eGFR > 60 mL/min/1.73 m^2^ in our study. Both DHEAS and UFC levels were shown to be decreased in our KTRs with suppressed baseline cortisol levels, confirming a complete downregulation of HPA axis activity in these KTRs.

Long-term glucocorticoid therapy, especially in high doses, is associated with adverse effects on lipid and glucose metabolism, as well as blood pressure regulation [51]. Whether low-dose maintenance glucocorticoids, commonly used in kidney transplantation, are associated with metabolic disorders is not clear [6]. Experience from steroid avoidance or withdrawal protocols in kidney transplantation suggests a potential cardiovascular benefit in recipients not receiving glucocorticoids, but with a greater risk for acute rejection episodes [5]. The prevalence of cardiovascular risk factors, such as hypertension, dyslipidemia, obesity and PTDM, in this cohort was high but comparable to earlier studies involving KTRs [52,53,54,55]. After adjusting for covariates, suppressed baseline cortisol levels were not associated with the presence of examined adverse outcomes in our cohort. An earlier study by de Vries et al. examined the relationship of 24 h UFC levels with metabolic syndrome in 563 stable KTRs. Suppressed UFC excretion was independently associated with BMI (*p* = 0.001), triglycerides (*p* = 0.001), diabetes (*p* = 0.005) and the number of antihypertensive medications (*p* = 0.003). However, participants in the study had a mean creatinine clearance of 62 ± 22 mL/min and received a higher-than-usual median daily prednisolone dose of 10.0 mg/day (IQR, 7.5–10.0) [30]. Data from different populations have indicated a potential dose-related risk of adrenal dysfunction and mortality [29]. The use of daily prednisone equivalents > 5 mg increased the risk of adverse metabolic events and all-cause mortality in rheumatoid arthritis patients, potentially as a result of higher cumulative glucocorticoid exposure [27,56]; this association was not observed with lower prednisolone doses [56].

Our study has several limitations to be considered. First, the lack of dynamic testing may have contributed to an underestimation of the prevalence of patients with glucocorticoid-induced HPA axis suppression, particularly those with baseline cortisol levels between 5 μg/dL and 10 μg/dL. A second potential limitation is that the inclusion of only KTRs with well-preserved renal graft function may have influenced the prevalence of HPA axis inhibition in our cohort compared to similar studies on kidney transplantation. However, this also serves as a strength, allowing for the extrapolation of our results to other glucocorticoid-treated populations. A third limitation is that there are many potential confounding factors for the increased prevalence of metabolic disorders in KTRs with glucocorticoid-induced HPA axis suppression. For instance, other immunosuppressive agents aside from glucocorticoids, such as calcineurin inhibitors and mTOR inhibitors, may also contribute to the development or worsening of adverse metabolic factors following transplantation [57]. Moreover, the cross-sectional design of our study limited the investigation of pre-existing adverse metabolic factors and the establishment of causal relationships. Lastly, it should be noted that the moderate sample size precluded robust conclusions regarding the potential association between glucocorticoid-induced HPA axis inhibition and metabolic disorders.

In conclusion, our study showed that HPA axis suppression is common in stable KTRs on low-dose maintenance glucocorticoid therapy, suggesting that even physiological doses of prednisolone may result in HPA axis activity inhibition. Increased age and a longer time since transplantation were more prevalent among KTRs with suppressed morning cortisol levels, indicating a greater cumulative glucocorticoid exposure. Baseline cortisol levels exhibited a positive correlation with other surrogate hormonal biomarkers, including ACTH, DHEAS and UFC levels. ACTH could serve as a complementary screening tool for the prediction of HPA axis inhibition in this population. An increased prevalence of cardiovascular risk factors is commonly observed among KTRs. Further investigation may determine whether biochemical evidence of glucocorticoid-induced HPA axis suppression is associated with a greater risk for adverse metabolic outcomes.

## Figures and Tables

**Table 1:** Demographic and clinical characteristics among all study participants and by baseline cortisol levels (<5 μg/dL and ≥5 μg/dL).

Characteristics	All Participants	F < 5 μg/dL	F ≥ 5 μg/dL	*p*-Value
Patients, *n* (%)	80	22 (27.5)	58 (72.5)	
Age (years), mean (±SD)	49.6 (14.8)	55.1 (14.0)	47.4 (14.6)	0.023
Male, *n* (%)	55 (68.8)	16 (72.7)	39 (67.2)	0.789
Female, *n* (%)	25 (31.2)	6 (27.3)	19 (32.8)	
BMI (kg/m^2^), median (IQR)	25.5 (22.0–29.1)	26.0 (23.2–29.7)	24.2 (21.6–28.4)	0.390
eGFR, (mL/min/1.73 m^2^), mean (±SD)	71.1 (5.7)	75.7 (4.8)	69.4 (5.9)	0.113
Living donor, *n* (%)	41 (51.3)	11 (50)	30 (51.7)	
Time from transplantation (months), median (IQR)	57 (24–102)	77 (36–158)	46 (23–100)	0.043
Dialysis vintage (years), median (IQR)	4 (2–9)	4 (2–7)	4 (1–9)	0.850
Tacrolimus, *n* (%)	68 (85.0)	16 (72.7)	52 (89.7)	0.064
Cyclosporine, *n* (%)	4 (5.0)	3 (13.6)	1 (1.7)	0.063
Everolimus, *n* (%)	12 (15.0)	6 (27.3)	6 (10.3)	0.083
Mycophenolic acid, *n* (%)	70 (87.5)	17 (77.3)	53 (91.4)	0.106
Prednisolone dose, mean (±SD)	5.00 (1.38)	5.20 (1.06)	5.12 (1.14)	0.712

F, serum cortisol level; BMI, body mass index; eGFR, estimated glomerular filtration rate; *p*-values obtained from chi-squared test, or two independent sample *t*-tests or Mann–Whitney U tests.

**Table 2:** Hormonal biomarkers among all study participants and by baseline cortisol levels (<5 μg/dL and ≥5 μg/dL).

	All Participants	F < 5 μg/dL	F ≥ 5 μg/dL	*p*-Value
Patients, *n* (%)	80	22 (27.5)	58 (72.5)	
Morning cortisol (μg/dL), mean (±SD)	9.0 (5.0)			
ACTH (pg/mL), median (IQR)	14.9 (9.3–29.9)	10.7 (6.2–12.9)	20.5 (11.7–38.0)	<0.001
DHEAS (μg/mL), median (IQR)	64.6 (25.7–107.0)	27.2 (17.2–50.5)	80.7 (37.0–25.7)	0.001
UFC (μg/24 h), median (IQR)	9.7 (5.2–21.6)	6.5 (4.1–10.8)	15.5 (6.3–29.2)	0.002

F, serum cortisol level; ACTH, adrenocorticotropic hormone; DHEAS, dehydroepiandrosterone-sulphate; UFC, 24 h urinary free cortisol; *p*-value obtained from Mann–Whitney U test.

**Table 3:** Adverse metabolic factors among all study participants and by baseline cortisol levels (<5 μg/dL and ≥5 μg/dL).

	All Participants	F < 5 μg/dL	F ≥ 5 μg/dL	*p*-Value
Patients, *n* (%)	80	22 (27.5)	58 (72.5)	
Hypertension, *n* (%)	58 (72.5)	21 (95.5)	37 (63.8)	0.006
Dyslipidemia, *n* (%)	50 (62.5)	13 (59.1)	37 (63.8)	0.698
Triglycerides (mg/dL), mean (±SD)	141.0 (50.8)	146.1 (53.8)	139.0 (50.0)	0.580
HDL-C (mg/dL), mean (±SD)	59.4 (14.5)	59.2 (17.3)	59.5 (13.5)	0.928
LDL-C (mg/dL), mean (±SD)	103.7 (34.6)	100.2 (31.0)	105.1 (36.0)	0.596
Central obesity, *n* (%)	36 (45.0)	14 (63.6)	22 (37.9)	0.039
PTDM, *n* (%)	11 (13.8)	4 (18.2)	7 (12.1)	0.497
Fasting glucose (mg/dL), mean (±SD)	102.8 (32.0)	107.0 (50.3)	101.2 (21.8)	0.470
HbA1c (%), mean (±SD)	5.7 (1.1)	6.0 (1.1)	5.6 (1.1)	0.141

F, serum cortisol level; HDL-C, high-density lipoprotein-cholesterol; LDL-C, low-density lipoprotein-cholesterol; PTDM, post-transplant diabetes mellitus; HbA1c, hemoglobin A1c; *p*-values obtained from chi-squared test or two independent sample *t*-tests.

**Table 4:** Association between glucocorticoid-induced HPA axis suppression and adverse metabolic factors.

Condition	Crude (OR, 95% CI)	Age and Sex Adjusted (OR, 95% CI)	Fully Adjusted (OR, 95% CI)
Hypertension	11.35 (1.42–90.72)	9.81 (1.17–82.29)	8.05 (0.86–75.25)
Dyslipidemia	0.82 (0.30–2.23)	0.78 (0.27–2.23)	0.74 (0.22–2.42)
Central obesity	2.86 (1.03–7.92)	2.28 (0.76–6.82)	3.58 (0.88–14.88)
PTDM	1.58 (0.41–6.07)	1.39 (0.34–5.73)	0.96 (0.20–4.60)

OR, odds ratio; CI, confidence interval; PTDM, post-transplant diabetes mellitus.

## Data Availability

The data that support the findings of this study are available from the corresponding author upon reasonable request.
